# Supplementary material for: 17-β-Estradiol—β-Cyclodextrin Complex as Solid: Synthesis, Structural and Physicochemical Characterization
Source: Molecules. 2023 Apr 26;28(9):3747. doi: 10.3390/molecules28093747 (PMC10180119; doi:10.3390/molecules28093747)

checkCIF/PLATON report

Structure factors have been supplied for datablock(s) cu_2022kbperst_0m

THIS REPORT IS FOR GUIDANCE ONLY. IF USED AS PART OF A REVIEW PROCEDURE FOR PUBLICATION, IT SHOULD NOT REPLACE THE EXPERTISE OF AN EXPERIENCED CRYSTALLOGRAPHIC REFEREE.

No syntax errors found. CIF dictionary Interpreting this report

Datablock: cu_2022kbperst_0m

Bond precision:	C-C = 0.0124 A		Wavelength=1.54178
Cell:	a=19.1245(15)	b=24.4180(18)	c=15.6004(11)
	alpha=90	beta=109.500(5)	gamma=90
Temperature:	100 K		

	Calculated	Reported
Volume	6867.2(9)	6867.2(9)
Space group	C 2	C 2
Hall group	C 2y	C 2y
Moiety formula	4(C42 H70 O35), 2(C18 H24 O2), O2, 40(O)	C51 H82 O46.5
Sum formula	C204 H328 O186	C51 H82 O46.50
Mr	5756.68	1439.16
Dx,g cm-3	1.392	1.392
Z	1	4
Mu (mm-1)	1.094	1.094
F000	3040.0	3040.0
F000'	3052.56	
h,k,lmax	22,29,18	22,29,18
Nref	12145[6229]	11945
Tmin,Tmax	0.737,0.867	0.593,0.754
Tmin'	0.615	

Correction method= # Reported T Limits: Tmin=0.593 Tmax=0.754
AbsCorr = MULTI-SCAN

Data completeness= 1.92/0.98 Theta(max)= 66.595

R(reflections)= 0.0952(10450) wR2(reflections)= 0.2688(11945)

S = 1.038 Npar= 950

The following ALERTS were generated. Each ALERT has the format
test-name_ALERT_alert-type_alert-level.
Click on the hyperlinks for more details of the test.

Alert level B

```
PLAT306_ALERT_2_B Isolated Oxygen Atom (H-atoms Missing ?) .......        O5W Check
PLAT306_ALERT_2_B Isolated Oxygen Atom (H-atoms Missing ?) .......        O6W Check
PLAT306_ALERT_2_B Isolated Oxygen Atom (H-atoms Missing ?) .......        O7W Check
PLAT306_ALERT_2_B Isolated Oxygen Atom (H-atoms Missing ?) .......       O10W Check
PLAT306_ALERT_2_B Isolated Oxygen Atom (H-atoms Missing ?) .......       O11W Check
PLAT306_ALERT_2_B Isolated Oxygen Atom (H-atoms Missing ?) .......       O12W Check
PLAT340_ALERT_3_B Low Bond Precision on  C-C Bonds ...............    0.01238 Ang.
PLAT430_ALERT_2_B Short Inter D...A Contact  O5W      ..O4WA     .       2.79 Ang.
                                                   x,y,z  =      1_555 Check
PLAT430_ALERT_2_B Short Inter D...A Contact  O5W      ..O11W     .       2.80 Ang.
                                                -1+x,y,z  =      1_455 Check
PLAT430_ALERT_2_B Short Inter D...A Contact  O6W      ..O2WA     .       2.66 Ang.
                                                   x,y,z  =      1_555 Check
PLAT430_ALERT_2_B Short Inter D...A Contact  O6W      ..O2WB     .       2.70 Ang.
                                                   x,y,z  =      1_555 Check
PLAT430_ALERT_2_B Short Inter D...A Contact  O6W      ..O7W      .       2.75 Ang.
                                                   x,y,z  =      1_555 Check
PLAT430_ALERT_2_B Short Inter D...A Contact  O6W      ..O4WA     .       2.80 Ang.
                                                 1+x,y,z  =      1_655 Check
PLAT430_ALERT_2_B Short Inter D...A Contact  O7W      ..O10W     .       2.77 Ang.
                                                 1+x,y,z  =      1_655 Check
PLAT430_ALERT_2_B Short Inter D...A Contact  O13W     ..O57_1    .       2.73 Ang.
                                                   x,y,z  =      1_555 Check
```

Alert level C

```
DIFMX02_ALERT_1_C  The maximum difference density is > 0.1*ZMAX*0.75
            The relevant atom site should be identified.
PLAT042_ALERT_1_C Calc. and Reported MoietyFormula Strings  Differ     Please Check
PLAT084_ALERT_3_C High wR2 Value (i.e. > 0.25) ...................       0.27 Report
PLAT089_ALERT_3_C Poor Data / Parameter Ratio (Zmax < 18) ........       6.54 Note
PLAT097_ALERT_2_C Large Reported Max.  (Positive) Residual Density       0.77 eA-3
PLAT202_ALERT_3_C Isotropic non-H Atoms in Anion/Solvent .........          6 Check
              O5W     O7W     O11W    O12W    O8WA    O9WA
PLAT220_ALERT_2_C NonSolvent   Resd 1  O    Ueq(max)/Ueq(min) Range        3.8 Ratio
PLAT222_ALERT_3_C NonSolvent Resd 1  H   Uiso(max)/Uiso(min) Range        5.1 Ratio
PLAT234_ALERT_4_C Large Hirshfeld Difference O61_1    --C61_1    .       0.21 Ang.
PLAT234_ALERT_4_C Large Hirshfeld Difference C56_1    --C66_1    .       0.16 Ang.
PLAT242_ALERT_2_C Low    'MainMol' Ueq as Compared to Neighbors of      C61_1 Check
PLAT260_ALERT_2_C Large Average Ueq of Residue Including       O3W      0.173 Check
PLAT430_ALERT_2_C Short Inter D...A Contact  O5W      ..O9WA     .       2.89 Ang.
                                         -1/2+x,1/2+y,z  =      3_455 Check
PLAT430_ALERT_2_C Short Inter D...A Contact  O6W      ..O4WB     .       2.86 Ang.
                                                 1+x,y,z  =      1_655 Check
PLAT911_ALERT_3_C Missing FCF Refl Between Thmin & STh/L=    0.595         17 Report
PLAT975_ALERT_2_C Check Calcd Resid. Dens.  0.64Ang From O9WB    .       0.45 eA-3
PLAT975_ALERT_2_C Check Calcd Resid. Dens.  1.02Ang From O9WA    .       0.41 eA-3
PLAT976_ALERT_2_C Check Calcd Resid. Dens.  0.49Ang From O11W    .      -0.42 eA-3
```

Alert level G

```
PLAT002_ALERT_2_G Number of Distance or Angle Restraints on AtSite         59 Note
PLAT003_ALERT_2_G Number of Uiso or Uij Restrained non-H Atoms ...         40 Report
PLAT007_ALERT_5_G Number of Unrefined Donor-H Atoms .............          27 Report
PLAT045_ALERT_1_G Calculated and Reported Z Differ by a Factor ...      0.250 Check
PLAT072_ALERT_2_G SHELXL First  Parameter in WGHT  Unusually Large       0.18 Report
PLAT083_ALERT_2_G SHELXL Second Parameter in WGHT  Unusually Large      15.85 Why ?
PLAT172_ALERT_4_G The CIF-Embedded .res File Contains DFIX Records         56 Report
PLAT173_ALERT_4_G The CIF-Embedded .res File Contains DANG Records         81 Report
PLAT174_ALERT_4_G The CIF-Embedded .res File Contains FLAT Records         18 Report
PLAT175_ALERT_4_G The CIF-Embedded .res File Contains SAME Records          2 Report
PLAT178_ALERT_4_G The CIF-Embedded .res File Contains SIMU Records          2 Report
PLAT300_ALERT_4_G Atom Site Occupancy of O64A_1     Constrained at        0.5 Check
PLAT300_ALERT_4_G Atom Site Occupancy of O64B_1     Constrained at        0.5 Check
PLAT300_ALERT_4_G Atom Site Occupancy of O67A_1     Constrained at        0.6 Check
PLAT300_ALERT_4_G Atom Site Occupancy of O67B_1     Constrained at        0.4 Check
PLAT300_ALERT_4_G Atom Site Occupancy of C64A_1     Constrained at        0.5 Check
PLAT300_ALERT_4_G Atom Site Occupancy of C64B_1     Constrained at        0.5 Check
PLAT300_ALERT_4_G Atom Site Occupancy of H64A_1     Constrained at        0.5 Check
PLAT300_ALERT_4_G Atom Site Occupancy of H64B_1     Constrained at        0.5 Check
PLAT300_ALERT_4_G Atom Site Occupancy of H64C_1     Constrained at        0.5 Check
PLAT300_ALERT_4_G Atom Site Occupancy of H64D_1     Constrained at        0.5 Check
PLAT300_ALERT_4_G Atom Site Occupancy of H64E_1     Constrained at        0.5 Check
PLAT300_ALERT_4_G Atom Site Occupancy of H64F_1     Constrained at        0.5 Check
PLAT300_ALERT_4_G Atom Site Occupancy of H67C_1     Constrained at        0.6 Check
PLAT300_ALERT_4_G Atom Site Occupancy of H67D_1     Constrained at        0.4 Check
PLAT300_ALERT_4_G Atom Site Occupancy of O2_2       Constrained at       0.25 Check
PLAT300_ALERT_4_G Atom Site Occupancy of O1_2       Constrained at       0.25 Check
PLAT300_ALERT_4_G Atom Site Occupancy of C11_2      Constrained at       0.25 Check
PLAT300_ALERT_4_G Atom Site Occupancy of C1_2       Constrained at       0.25 Check
PLAT300_ALERT_4_G Atom Site Occupancy of C6_2       Constrained at       0.25 Check
PLAT300_ALERT_4_G Atom Site Occupancy of C8_2       Constrained at       0.25 Check
PLAT300_ALERT_4_G Atom Site Occupancy of C4_2       Constrained at       0.25 Check
PLAT300_ALERT_4_G Atom Site Occupancy of C12_2      Constrained at       0.25 Check
PLAT300_ALERT_4_G Atom Site Occupancy of C15_2      Constrained at       0.25 Check
PLAT300_ALERT_4_G Atom Site Occupancy of C17_2      Constrained at       0.25 Check
PLAT300_ALERT_4_G Atom Site Occupancy of C18_2      Constrained at       0.25 Check
PLAT300_ALERT_4_G Atom Site Occupancy of C16_2      Constrained at       0.25 Check
PLAT300_ALERT_4_G Atom Site Occupancy of C14_2      Constrained at       0.25 Check
PLAT300_ALERT_4_G Atom Site Occupancy of C13_2      Constrained at       0.25 Check
PLAT300_ALERT_4_G Atom Site Occupancy of C10_2      Constrained at       0.25 Check
PLAT300_ALERT_4_G Atom Site Occupancy of C3_2       Constrained at       0.25 Check
PLAT300_ALERT_4_G Atom Site Occupancy of C2_2       Constrained at       0.25 Check
PLAT300_ALERT_4_G Atom Site Occupancy of C7_2       Constrained at       0.25 Check
PLAT300_ALERT_4_G Atom Site Occupancy of C9_2       Constrained at       0.25 Check
PLAT300_ALERT_4_G Atom Site Occupancy of C5_2       Constrained at       0.25 Check
PLAT300_ALERT_4_G Atom Site Occupancy of H11A_2     Constrained at       0.25 Check
PLAT300_ALERT_4_G Atom Site Occupancy of H11B_2     Constrained at       0.25 Check
PLAT300_ALERT_4_G Atom Site Occupancy of H11C_2     Constrained at       0.25 Check
PLAT300_ALERT_4_G Atom Site Occupancy of H6A_2      Constrained at       0.25 Check
PLAT300_ALERT_4_G Atom Site Occupancy of H6B_2      Constrained at       0.25 Check
PLAT300_ALERT_4_G Atom Site Occupancy of H8A_2      Constrained at       0.25 Check
PLAT300_ALERT_4_G Atom Site Occupancy of H8B_2      Constrained at       0.25 Check
PLAT300_ALERT_4_G Atom Site Occupancy of H4_2       Constrained at       0.25 Check
PLAT300_ALERT_4_G Atom Site Occupancy of H15_2      Constrained at       0.25 Check
PLAT300_ALERT_4_G Atom Site Occupancy of H17_2      Constrained at       0.25 Check
PLAT300_ALERT_4_G Atom Site Occupancy of H2K_2      Constrained at       0.25 Check
```

```
PLAT300_ALERT_4_G Atom Site Occupancy of H16_2      Constrained at       0.25 Check
PLAT300_ALERT_4_G Atom Site Occupancy of H13A_2     Constrained at       0.25 Check
PLAT300_ALERT_4_G Atom Site Occupancy of H13B_2     Constrained at       0.25 Check
PLAT300_ALERT_4_G Atom Site Occupancy of H10A_2     Constrained at       0.25 Check
PLAT300_ALERT_4_G Atom Site Occupancy of H10B_2     Constrained at       0.25 Check
PLAT300_ALERT_4_G Atom Site Occupancy of H3_2       Constrained at       0.25 Check
PLAT300_ALERT_4_G Atom Site Occupancy of H2_2       Constrained at       0.25 Check
PLAT300_ALERT_4_G Atom Site Occupancy of H7A_2      Constrained at       0.25 Check
PLAT300_ALERT_4_G Atom Site Occupancy of H7B_2      Constrained at       0.25 Check
PLAT300_ALERT_4_G Atom Site Occupancy of H9A_2      Constrained at       0.25 Check
PLAT300_ALERT_4_G Atom Site Occupancy of H9B_2      Constrained at       0.25 Check
PLAT300_ALERT_4_G Atom Site Occupancy of H5_2       Constrained at       0.25 Check
PLAT300_ALERT_4_G Atom Site Occupancy of H1_2       Constrained at       0.25 Check
PLAT300_ALERT_4_G Atom Site Occupancy of O2_3       Constrained at       0.25 Check
PLAT300_ALERT_4_G Atom Site Occupancy of O1_3       Constrained at       0.25 Check
PLAT300_ALERT_4_G Atom Site Occupancy of C16_3      Constrained at       0.25 Check
PLAT300_ALERT_4_G Atom Site Occupancy of C18_3      Constrained at       0.25 Check
PLAT300_ALERT_4_G Atom Site Occupancy of C17_3      Constrained at       0.25 Check
PLAT300_ALERT_4_G Atom Site Occupancy of C15_3      Constrained at       0.25 Check
PLAT300_ALERT_4_G Atom Site Occupancy of C12_3      Constrained at       0.25 Check
PLAT300_ALERT_4_G Atom Site Occupancy of C14_3      Constrained at       0.25 Check
PLAT300_ALERT_4_G Atom Site Occupancy of C13_3      Constrained at       0.25 Check
PLAT300_ALERT_4_G Atom Site Occupancy of C10_3      Constrained at       0.25 Check
PLAT300_ALERT_4_G Atom Site Occupancy of C3_3       Constrained at       0.25 Check
PLAT300_ALERT_4_G Atom Site Occupancy of C2_3       Constrained at       0.25 Check
PLAT300_ALERT_4_G Atom Site Occupancy of C7_3       Constrained at       0.25 Check
PLAT300_ALERT_4_G Atom Site Occupancy of C9_3       Constrained at       0.25 Check
PLAT300_ALERT_4_G Atom Site Occupancy of C5_3       Constrained at       0.25 Check
PLAT300_ALERT_4_G Atom Site Occupancy of C1_3       Constrained at       0.25 Check
PLAT300_ALERT_4_G Atom Site Occupancy of C11_3      Constrained at       0.25 Check
PLAT300_ALERT_4_G Atom Site Occupancy of C6_3       Constrained at       0.25 Check
PLAT300_ALERT_4_G Atom Site Occupancy of C4_3       Constrained at       0.25 Check
PLAT300_ALERT_4_G Atom Site Occupancy of C8_3       Constrained at       0.25 Check
PLAT300_ALERT_4_G Atom Site Occupancy of H2_3       Constrained at       0.25 Check
PLAT300_ALERT_4_G Atom Site Occupancy of H16_3      Constrained at       0.25 Check
PLAT300_ALERT_4_G Atom Site Occupancy of H17_3      Constrained at       0.25 Check
PLAT300_ALERT_4_G Atom Site Occupancy of H15_3      Constrained at       0.25 Check
PLAT300_ALERT_4_G Atom Site Occupancy of H13A_3     Constrained at       0.25 Check
PLAT300_ALERT_4_G Atom Site Occupancy of H13B_3     Constrained at       0.25 Check
PLAT300_ALERT_4_G Atom Site Occupancy of H10A_3     Constrained at       0.25 Check
PLAT300_ALERT_4_G Atom Site Occupancy of H10B_3     Constrained at       0.25 Check
PLAT300_ALERT_4_G Atom Site Occupancy of H3_3       Constrained at       0.25 Check
PLAT300_ALERT_4_G Atom Site Occupancy of H2A_3      Constrained at       0.25 Check
PLAT300_ALERT_4_G Atom Site Occupancy of H7A_3      Constrained at       0.25 Check
PLAT300_ALERT_4_G Atom Site Occupancy of H7B_3      Constrained at       0.25 Check
PLAT300_ALERT_4_G Atom Site Occupancy of H9A_3      Constrained at       0.25 Check
PLAT300_ALERT_4_G Atom Site Occupancy of H9B_3      Constrained at       0.25 Check
PLAT300_ALERT_4_G Atom Site Occupancy of H5_3       Constrained at       0.25 Check
PLAT300_ALERT_4_G Atom Site Occupancy of H1_3       Constrained at       0.25 Check
PLAT300_ALERT_4_G Atom Site Occupancy of H11A_3     Constrained at       0.25 Check
PLAT300_ALERT_4_G Atom Site Occupancy of H11B_3     Constrained at       0.25 Check
PLAT300_ALERT_4_G Atom Site Occupancy of H11C_3     Constrained at       0.25 Check
PLAT300_ALERT_4_G Atom Site Occupancy of H6A_3      Constrained at       0.25 Check
PLAT300_ALERT_4_G Atom Site Occupancy of H6B_3      Constrained at       0.25 Check
PLAT300_ALERT_4_G Atom Site Occupancy of H4_3       Constrained at       0.25 Check
PLAT300_ALERT_4_G Atom Site Occupancy of H8A_3      Constrained at       0.25 Check
PLAT300_ALERT_4_G Atom Site Occupancy of H8B_3      Constrained at       0.25 Check
```

```
PLAT300_ALERT_4_G Atom Site Occupancy of O1W        Constrained at        0.5 Check
PLAT300_ALERT_4_G Atom Site Occupancy of O3W        Constrained at        0.5 Check
PLAT300_ALERT_4_G Atom Site Occupancy of O13W       Constrained at        0.5 Check
PLAT300_ALERT_4_G Atom Site Occupancy of O2WA       Constrained at        0.5 Check
PLAT300_ALERT_4_G Atom Site Occupancy of O2WB       Constrained at        0.5 Check
PLAT300_ALERT_4_G Atom Site Occupancy of O4WA       Constrained at        0.5 Check
PLAT300_ALERT_4_G Atom Site Occupancy of O4WB       Constrained at        0.5 Check
PLAT300_ALERT_4_G Atom Site Occupancy of O8WA       Constrained at        0.7 Check
PLAT300_ALERT_4_G Atom Site Occupancy of O9WA       Constrained at        0.6 Check
PLAT300_ALERT_4_G Atom Site Occupancy of Ow8B       Constrained at        0.3 Check
PLAT300_ALERT_4_G Atom Site Occupancy of O9WB       Constrained at        0.4 Check
PLAT301_ALERT_3_G Main Residue  Disorder .............(Resd  1  )          4% Note
PLAT302_ALERT_4_G Anion/Solvent/Minor-Residue Disorder (Resd  2  )       100% Note
PLAT302_ALERT_4_G Anion/Solvent/Minor-Residue Disorder (Resd  3  )       100% Note
PLAT302_ALERT_4_G Anion/Solvent/Minor-Residue Disorder (Resd  4  )       100% Note
PLAT302_ALERT_4_G Anion/Solvent/Minor-Residue Disorder (Resd  5  )       100% Note
PLAT302_ALERT_4_G Anion/Solvent/Minor-Residue Disorder (Resd 12  )       100% Note
PLAT302_ALERT_4_G Anion/Solvent/Minor-Residue Disorder (Resd 13  )       100% Note
PLAT302_ALERT_4_G Anion/Solvent/Minor-Residue Disorder (Resd 14  )       100% Note
PLAT302_ALERT_4_G Anion/Solvent/Minor-Residue Disorder (Resd 15  )       100% Note
PLAT302_ALERT_4_G Anion/Solvent/Minor-Residue Disorder (Resd 16  )       100% Note
PLAT302_ALERT_4_G Anion/Solvent/Minor-Residue Disorder (Resd 17  )       100% Note
PLAT302_ALERT_4_G Anion/Solvent/Minor-Residue Disorder (Resd 18  )       100% Note
PLAT302_ALERT_4_G Anion/Solvent/Minor-Residue Disorder (Resd 19  )       100% Note
PLAT302_ALERT_4_G Anion/Solvent/Minor-Residue Disorder (Resd 20  )       100% Note
PLAT304_ALERT_4_G Non-Integer Number of Atoms in ..... (Resd  5  )       0.50 Check
PLAT304_ALERT_4_G Non-Integer Number of Atoms in ..... (Resd  9  )       0.50 Check
PLAT304_ALERT_4_G Non-Integer Number of Atoms in ..... (Resd 11  )       0.50 Check
PLAT304_ALERT_4_G Non-Integer Number of Atoms in ..... (Resd 12  )       0.50 Check
PLAT304_ALERT_4_G Non-Integer Number of Atoms in ..... (Resd 13  )       0.50 Check
PLAT304_ALERT_4_G Non-Integer Number of Atoms in ..... (Resd 14  )       0.50 Check
PLAT304_ALERT_4_G Non-Integer Number of Atoms in ..... (Resd 15  )       0.50 Check
PLAT304_ALERT_4_G Non-Integer Number of Atoms in ..... (Resd 16  )       0.50 Check
PLAT304_ALERT_4_G Non-Integer Number of Atoms in ..... (Resd 17  )       0.70 Check
PLAT304_ALERT_4_G Non-Integer Number of Atoms in ..... (Resd 18  )       0.60 Check
PLAT304_ALERT_4_G Non-Integer Number of Atoms in ..... (Resd 19  )       0.30 Check
PLAT304_ALERT_4_G Non-Integer Number of Atoms in ..... (Resd 20  )       0.40 Check
PLAT311_ALERT_2_G Isolated Disordered Oxygen Atom (No H's ?) .....        O1W Check
PLAT311_ALERT_2_G Isolated Disordered Oxygen Atom (No H's ?) .....        O3W Check
PLAT311_ALERT_2_G Isolated Disordered Oxygen Atom (No H's ?) .....       O13W Check
PLAT311_ALERT_2_G Isolated Disordered Oxygen Atom (No H's ?) .....       O2WA Check
PLAT311_ALERT_2_G Isolated Disordered Oxygen Atom (No H's ?) .....       O2WB Check
PLAT311_ALERT_2_G Isolated Disordered Oxygen Atom (No H's ?) .....       O4WA Check
PLAT311_ALERT_2_G Isolated Disordered Oxygen Atom (No H's ?) .....       O4WB Check
PLAT311_ALERT_2_G Isolated Disordered Oxygen Atom (No H's ?) .....       O8WA Check
PLAT311_ALERT_2_G Isolated Disordered Oxygen Atom (No H's ?) .....       O9WA Check
PLAT311_ALERT_2_G Isolated Disordered Oxygen Atom (No H's ?) .....       Ow8B Check
PLAT311_ALERT_2_G Isolated Disordered Oxygen Atom (No H's ?) .....       O9WB Check
PLAT411_ALERT_2_G Short Inter H...H Contact  H35_1     ..H8B_2    .      2.11 Ang.
                                                    x,y,z  =      1_555 Check
PLAT414_ALERT_2_G Short Intra D-H..H-X       H67A_1    ..H67D_1   .      1.00 Ang.
                                                    x,y,z  =      1_555 Check
PLAT414_ALERT_2_G Short Intra D-H..H-X       H67B_1    ..H67C_1   .      2.08 Ang.
                                                    x,y,z  =      1_555 Check
PLAT415_ALERT_2_G Short Inter D-H..H-X       H61_1     ..H64A_1   .      1.86 Ang.
                                                1-x,y,1-z  =      2_656 Check
PLAT415_ALERT_2_G Short Inter D-H..H-X       H66A_1    ..H67D_1   .      1.75 Ang.
```

```
                                                   1-x,y,1-z  =      2_656 Check
PLAT432_ALERT_2_G Short Inter X...Y Contact  O13W     ..C66_1    .        3.02 Ang.
                                                   1-x,y,1-z  =      2_656 Check
PLAT650_ALERT_4_G SWAT Instruction Used to Model Solvent Disorder           ! Report
PLAT720_ALERT_4_G Number of Unusual/Non-Standard Labels ..........        250 Note
PLAT789_ALERT_4_G Atoms with Negative _atom_site_disorder_group  #         88 Check
PLAT791_ALERT_4_G Model has Chirality at C11_1       (Sohnke SpGr)          S Verify
PLAT791_ALERT_4_G Model has Chirality at C21_1       (Sohnke SpGr)          R Verify
PLAT791_ALERT_4_G Model has Chirality at C31_1       (Sohnke SpGr)          S Verify
PLAT791_ALERT_4_G Model has Chirality at C41_1       (Sohnke SpGr)          S Verify
PLAT791_ALERT_4_G Model has Chirality at C51_1       (Sohnke SpGr)          R Verify
PLAT791_ALERT_4_G Model has Chirality at C12_1       (Sohnke SpGr)          S Verify
PLAT791_ALERT_4_G Model has Chirality at C22_1       (Sohnke SpGr)          R Verify
PLAT791_ALERT_4_G Model has Chirality at C32_1       (Sohnke SpGr)          S Verify
PLAT791_ALERT_4_G Model has Chirality at C42_1       (Sohnke SpGr)          S Verify
PLAT791_ALERT_4_G Model has Chirality at C52_1       (Sohnke SpGr)          R Verify
PLAT791_ALERT_4_G Model has Chirality at C13_1       (Sohnke SpGr)          S Verify
PLAT791_ALERT_4_G Model has Chirality at C23_1       (Sohnke SpGr)          R Verify
PLAT791_ALERT_4_G Model has Chirality at C33_1       (Sohnke SpGr)          S Verify
PLAT791_ALERT_4_G Model has Chirality at C43_1       (Sohnke SpGr)          S Verify
PLAT791_ALERT_4_G Model has Chirality at C53_1       (Sohnke SpGr)          R Verify
PLAT791_ALERT_4_G Model has Chirality at C24_1       (Sohnke SpGr)          R Verify
PLAT791_ALERT_4_G Model has Chirality at C34_1       (Sohnke SpGr)          S Verify
PLAT791_ALERT_4_G Model has Chirality at C15_1       (Sohnke SpGr)          S Verify
PLAT791_ALERT_4_G Model has Chirality at C25_1       (Sohnke SpGr)          R Verify
PLAT791_ALERT_4_G Model has Chirality at C35_1       (Sohnke SpGr)          S Verify
PLAT791_ALERT_4_G Model has Chirality at C45_1       (Sohnke SpGr)          S Verify
PLAT791_ALERT_4_G Model has Chirality at C55_1       (Sohnke SpGr)          R Verify
PLAT791_ALERT_4_G Model has Chirality at C16_1       (Sohnke SpGr)          S Verify
PLAT791_ALERT_4_G Model has Chirality at C26_1       (Sohnke SpGr)          R Verify
PLAT791_ALERT_4_G Model has Chirality at C36_1       (Sohnke SpGr)          S Verify
PLAT791_ALERT_4_G Model has Chirality at C46_1       (Sohnke SpGr)          S Verify
PLAT791_ALERT_4_G Model has Chirality at C56_1       (Sohnke SpGr)          R Verify
PLAT791_ALERT_4_G Model has Chirality at C27_1       (Sohnke SpGr)          R Verify
PLAT791_ALERT_4_G Model has Chirality at C37_1       (Sohnke SpGr)          S Verify
PLAT791_ALERT_4_G Model has Chirality at C1_2        (Sohnke SpGr)          S Verify
PLAT791_ALERT_4_G Model has Chirality at C4_2        (Sohnke SpGr)          S Verify
PLAT791_ALERT_4_G Model has Chirality at C3_2        (Sohnke SpGr)          R Verify
PLAT791_ALERT_4_G Model has Chirality at C2_2        (Sohnke SpGr)          S Verify
PLAT791_ALERT_4_G Model has Chirality at C5_2        (Sohnke SpGr)          S Verify
PLAT791_ALERT_4_G Model has Chirality at C3_3        (Sohnke SpGr)          R Verify
PLAT791_ALERT_4_G Model has Chirality at C2_3        (Sohnke SpGr)          S Verify
PLAT791_ALERT_4_G Model has Chirality at C5_3        (Sohnke SpGr)          S Verify
PLAT791_ALERT_4_G Model has Chirality at C1_3        (Sohnke SpGr)          S Verify
PLAT791_ALERT_4_G Model has Chirality at C4_3        (Sohnke SpGr)          S Verify
PLAT811_ALERT_5_G No ADDSYM Analysis: Too Many Excluded Atoms ....          ! Info
PLAT860_ALERT_3_G Number of Least-Squares Restraints ............         202 Note
PLAT883_ALERT_1_G No Info/Value for _atom_sites_solution_primary .     Please Do !
PLAT909_ALERT_3_G Percentage of I>2sig(I) Data at Theta(Max) Still        59% Note
PLAT933_ALERT_2_G Number of HKL-OMIT Records in Embedded .res File         17 Note
PLAT978_ALERT_2_G Number C-C Bonds with Positive Residual Density.          0 Info
```

---

0 **ALERT level A** = Most likely a serious problem - resolve or explain
15 **ALERT level B** = A potentially serious problem, consider carefully
18 **ALERT level C** = Check. Ensure it is not caused by an omission or oversight
215 **ALERT level G** = General information/check it is not something unexpected

```
  4 ALERT type 1 CIF construction/syntax error, inconsistent or missing data
 46 ALERT type 2 Indicator that the structure model may be wrong or deficient
  9 ALERT type 3 Indicator that the structure quality may be low
187 ALERT type 4 Improvement, methodology, query or suggestion
  2 ALERT type 5 Informative message, check
```

It is advisable to attempt to resolve as many as possible of the alerts in all categories. Often the minor alerts point to easily fixed oversights, errors and omissions in your CIF or refinement strategy, so attention to these fine details can be worthwhile. In order to resolve some of the more serious problems it may be necessary to carry out additional measurements or structure refinements. However, the purpose of your study may justify the reported deviations and the more serious of these should normally be commented upon in the discussion or experimental section of a paper or in the "special_details" fields of the CIF. checkCIF was carefully designed to identify outliers and unusual parameters, but every test has its limitations and alerts that are not important in a particular case may appear. Conversely, the absence of alerts does not guarantee there are no aspects of the results needing attention. It is up to the individual to critically assess their own results and, if necessary, seek expert advice.

Publication of your CIF in IUCr journals

A basic structural check has been run on your CIF. These basic checks will be run on all CIFs submitted for publication in IUCr journals (*Acta Crystallographica*, *Journal of Applied Crystallography*, *Journal of Synchrotron Radiation*); however, if you intend to submit to *Acta Crystallographica Section C* or *E* or *IUCrData*, you should make sure that full publication checks are run on the final version of your CIF prior to submission.

Publication of your CIF in other journals

Please refer to the *Notes for Authors* of the relevant journal for any special instructions relating to CIF submission.

PLATON version of 28/11/2022; check.def file version of 28/11/2022

Datablock cu_2022kbperst_0m - ellipsoid plot

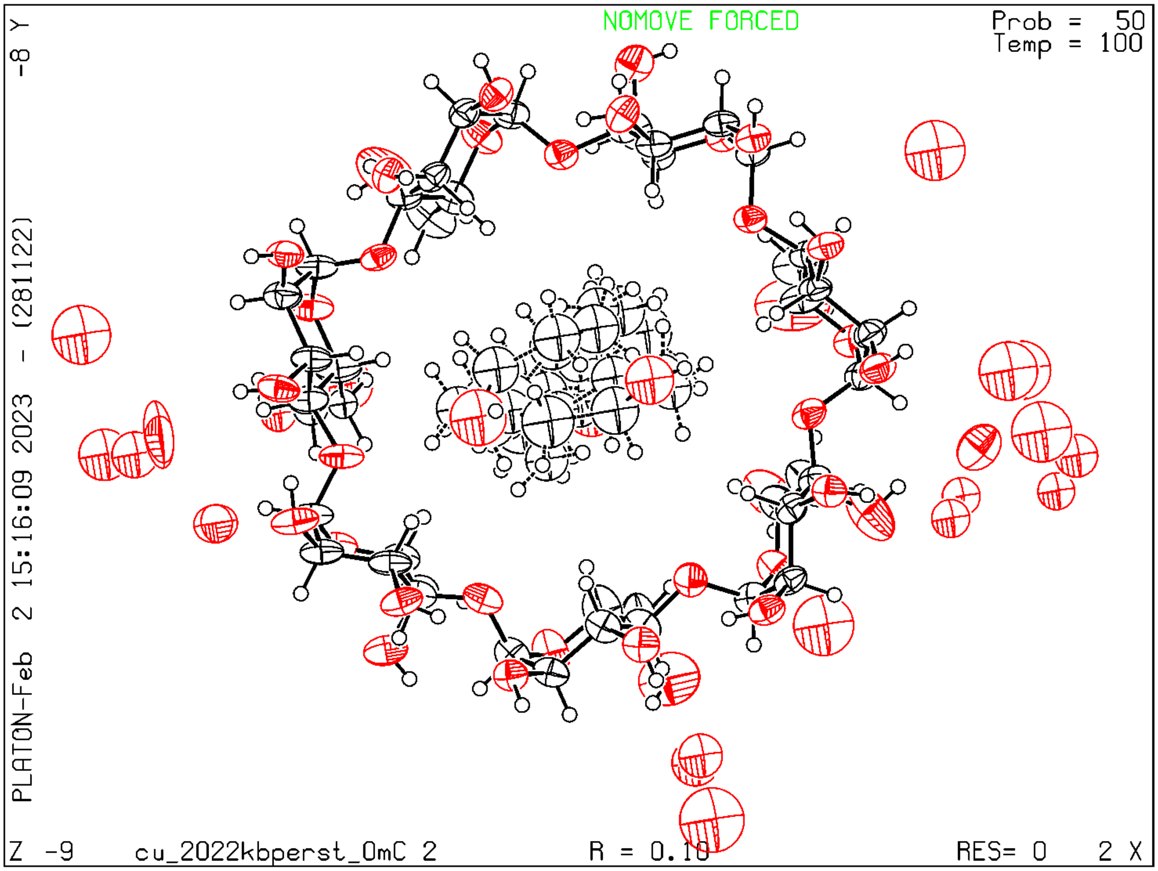

Supplement: Supplementary file 1 [file molecules-28-03747-s001.zip › checkcif-1.pdf]
