# Supplementary material for: 17-β-Estradiol—β-Cyclodextrin Complex as Solid: Synthesis, Structural and Physicochemical Characterization
Source: Molecules. 2023 Apr 26;28(9):3747. doi: 10.3390/molecules28093747 (PMC10180119; doi:10.3390/molecules28093747)
Supplement: Supplementary file 1 [file molecules-28-03747-s001.zip › molecules-2348482-supplementary.pdf]

Table S1: Geometric parameters of the *β*-CD host molecule in the Est/*β*-CD crystal structure.

The glucosidic O4*n* atoms form a nearly regular heptagon and their mean plane forms an angle of 9.76(7)° with the crystallographic *ab* plane. The O64 and O67 atoms of the primary rim hydroxyls are disordered over two sites (A and B). The torsion angle *t* (O5*n*-C5*n*-C6*n*-O6*n*) values for these residues indicate that they adopt both gauche-gauche (*gg*) and gauche-trans (*gt*) conformations in the crystal lattice. All remaining glucose units, with the exception of G1 (*gt*) adopt the *gg* conformation, their O6*n* atoms point outwards the CD cavity, aiding the H-bond formation between the CD macrocycle and the neighboring water molecules.

	D (Å)	***D*K** (Å)	***d*** (Å)	***Φ*n** (°)	***Φ*K** (°)	***τ*** (°)	***t*** (°)	***C***
**Residue number**								
***n*=1**	4.281(3)	5.155(3)	0.055(4)	125.610(18)	50.375(2)	10.5737(12)	61.659(5)	*gt*
***n*=2**	4.485(3)	4.889(4)	0.004(3)	131.860(5)	54.123(4)	4.1786(4)	-65.538(2)	*gg*
***n*=3**	4.333(3)	4.968(3)	-0.0711(5)	129.610(2)	50.380(4)	11.5198(11)	-60.109(5)	*gg*
***n*=4**	4.295(2)	5.201(4)	0.044(3)	124.807(5)	49.715(2)	12.8987(13)	-64.482(2) (siteA: 0.5)	*gg*
							76.014(4) (siteB: 0.5)	*gt*
***n*=5**	4.455(4)	5.007(3)	0.035(3)	129.554(3)	53.428(5)	8.248(8)	-61.453(5)	*gg*
***n*=6**	4.362(2)	4.902(3)	-0.046(4)	130.753(4)	51.767(4)	12.6513(13)	-69.684(5)	*gg*
***n*=7**	4.347(3)	5.084(4)	-0.0199(15)	127.690(4)	50.247(5)	12.4335(11)	-57.571(4) (siteA: 0.6)	*gg*
							70.7795(13) (siteA: 0.4)	*gt*

(**a**) *D*: O4*n*…O4(*n*+1) distances; (**b**) *D*K: K…O4 distances of the approximate center K of the O4*n* heptagon from the O4*n* atoms; (**c**) *d*: deviations of the O4*n* atoms from their least-squares plane; (**d**) *Φ*n: O4(*n*-1)…O4*n*…O4(*n*+1) angles; (**e**) *Φ*K: O4*n*…K…O4(*n*+1) angles; (**f**) *τ*: tilt angles between the optimum O4*n* plane and the mean plane of the O4(*n*-1). . .C1*n* . . .C4*n*. . .O4*n* atoms; (**g**) *t*: O5*n* . . . C5*n* . . . C6*n* . . . O6*n* torsion angles; (**h**) *C*: conformation of the primary methoxy groups. All distances are given in Å and angles in (°). The O4*n* atoms plane equation is: 0.169*X -0.008*Y -0.986*Z -10.399 = 0

Table S2. Main H-bonds in the crystal structure of Est/β-CD inclusion complex as found by Olex2 with a maximum distance of 2.9 Å and a minimum angle of 120°.

Atom 1	Atom 2	Distance (Å)	Atom 1	Atom 2	Distance (Å)
O2 (S1)	O61 (1-x,y,1-z)	2.92(6)	O2 (S2)	O61 (1-x,y,1-z)	2.62(6)
O2 (S1)	O67B (1-x,y,1-z)	2.81(6)	O2 (S2)	O2 (S1) (1-x,y,1-z)	2.83(7)
O2 (S1)	O2 (S2) (1-x,y,1-z)	2.83(7)	O2 (S2)	O2 (S2) (1-x,y,1-z)	2.66(11)
O21	O1W	2.796(2)	O31	O22	2.779(3)
O61	O64B (1-x,y,1-z)	2.887(2)	O32	O23	2.763(3)
O22	O27 (3/2x,1/2+y,2-z)	2.746(2)	O25	O34	2.866(2)
O62	O65 (1/2+x,1/2+y,z)	2.8198(17)	O35	O26	2.816(2)
O23	O8WA (-1/2+x,1/2+y,z)	2.659(1)			
O23	OW8B (-1/2+x,1/2+y,z)	2.839(1)			
O33	O32 (1-x,y,2-z)	2.776(2)			
O24	O9WA (-1/2+x,1/2+y,z)	2.828(2)			
O64A	O12W (-1/2+x,1/2+y,z)	2.791(2)			
O64B	O4WB (-x,y,1-z)	2.835(2)			
O65	O63 (1/2-x,-1/2+y,1-z)	2.765(1)			
O26	O9WB (1-x,y,2-z)	2.7819(16)			
O36	O9WA	2.816(2)			
O66	O11W (3/2-x,-1/2+y,z)	2.822(2)			
O27	O36	2.792(2)			
O37	O8WA	2.844(2)			
O37	OW8B	2.669(2)			
O67A	O6W	2.772(2)			

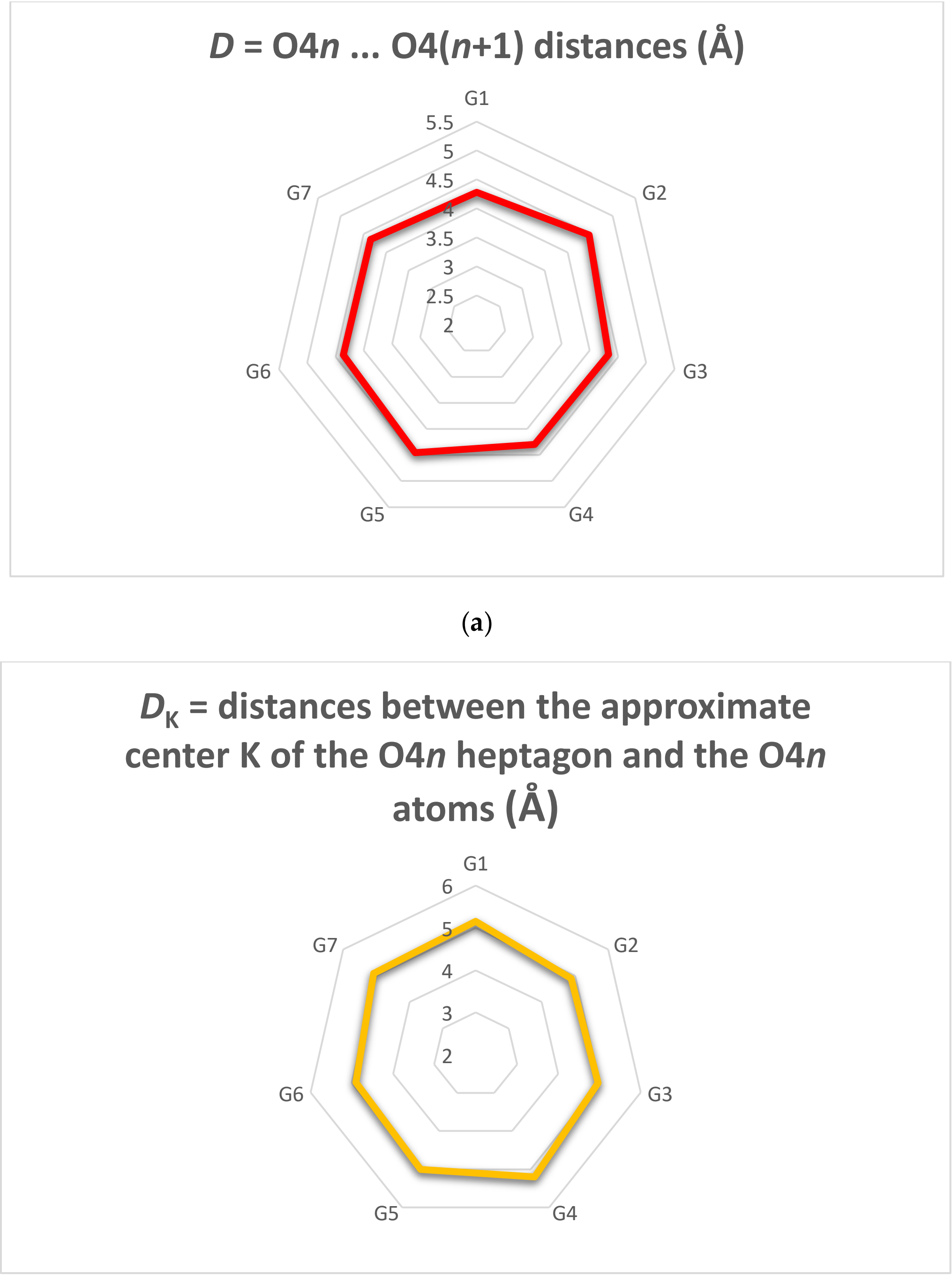
D = O4n ... O4(n+1) distances (Å)
G1
G2
G3
G4
G5
G6
G7
5.5
5
4.5
4
3.5
3
2.5
2
DK = distances between the approximate center K of the O4n heptagon and the O4n atoms (Å)
6
5
4
3
2

(a)

(b)

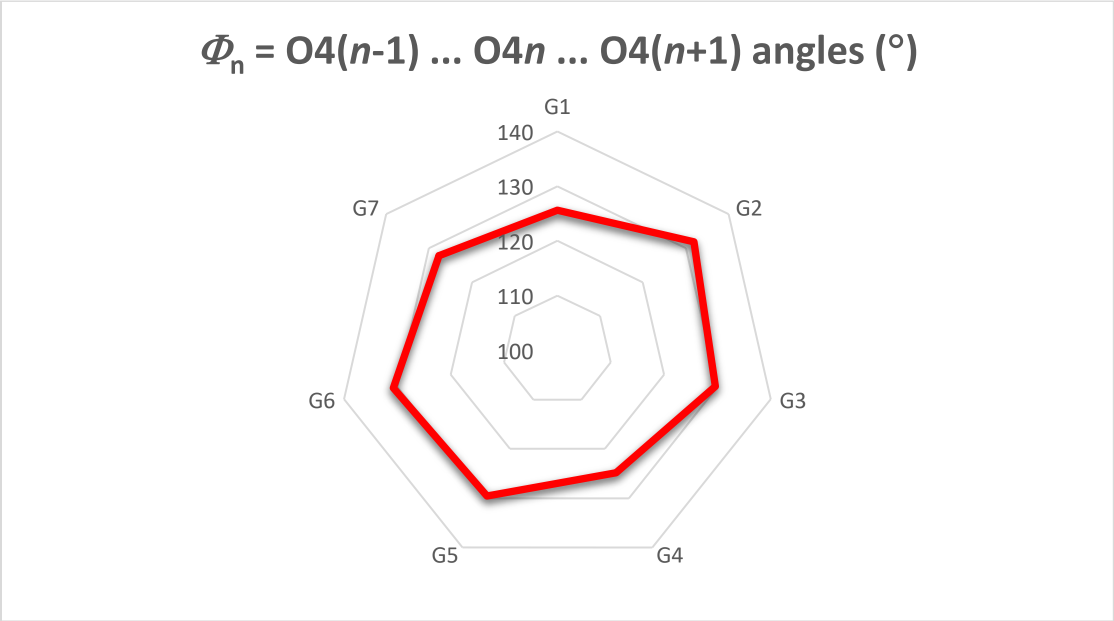

(**c**)

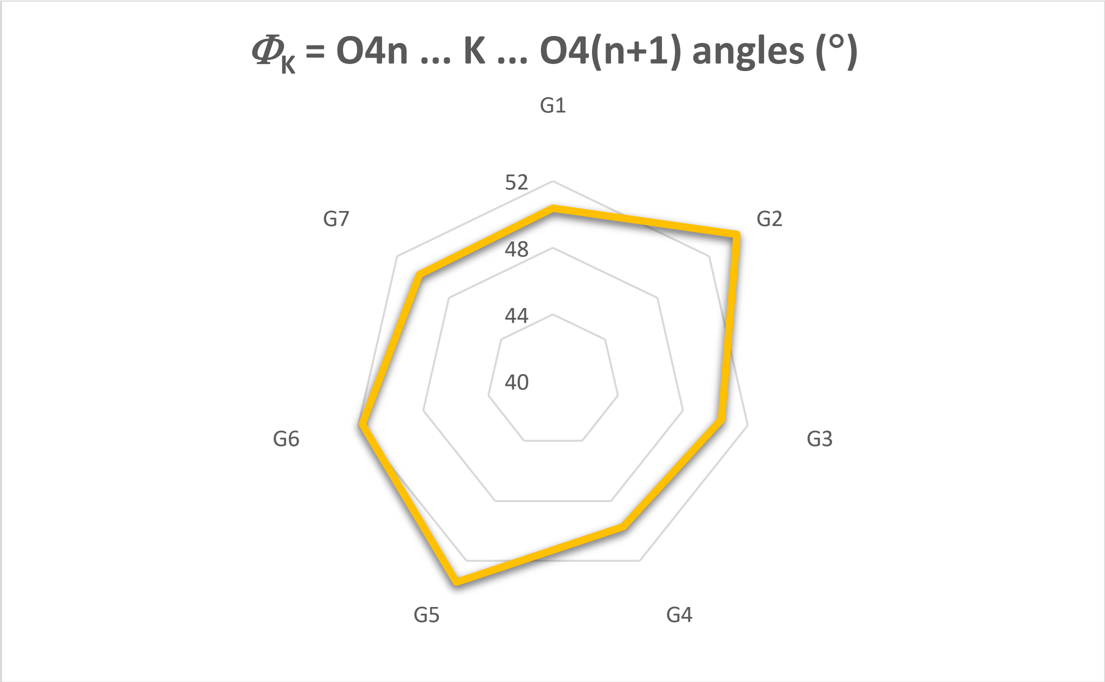

(**d**)

Figure S1: Radar plots of distances and angles as defined in Table S1. (**a**) *D*: O4*n*…O4(*n*+1) distances; (**b**) D_K: K…O4 distances of the approximate center K of the O4*n* heptagon from the O4*n* atoms; (**c**) Φ_n: O4(*n*-1)…O4*n* … O4(*n*+1) angles; (**d**) Φ_K: O4*n*… K…O4(*n*+1) angles.

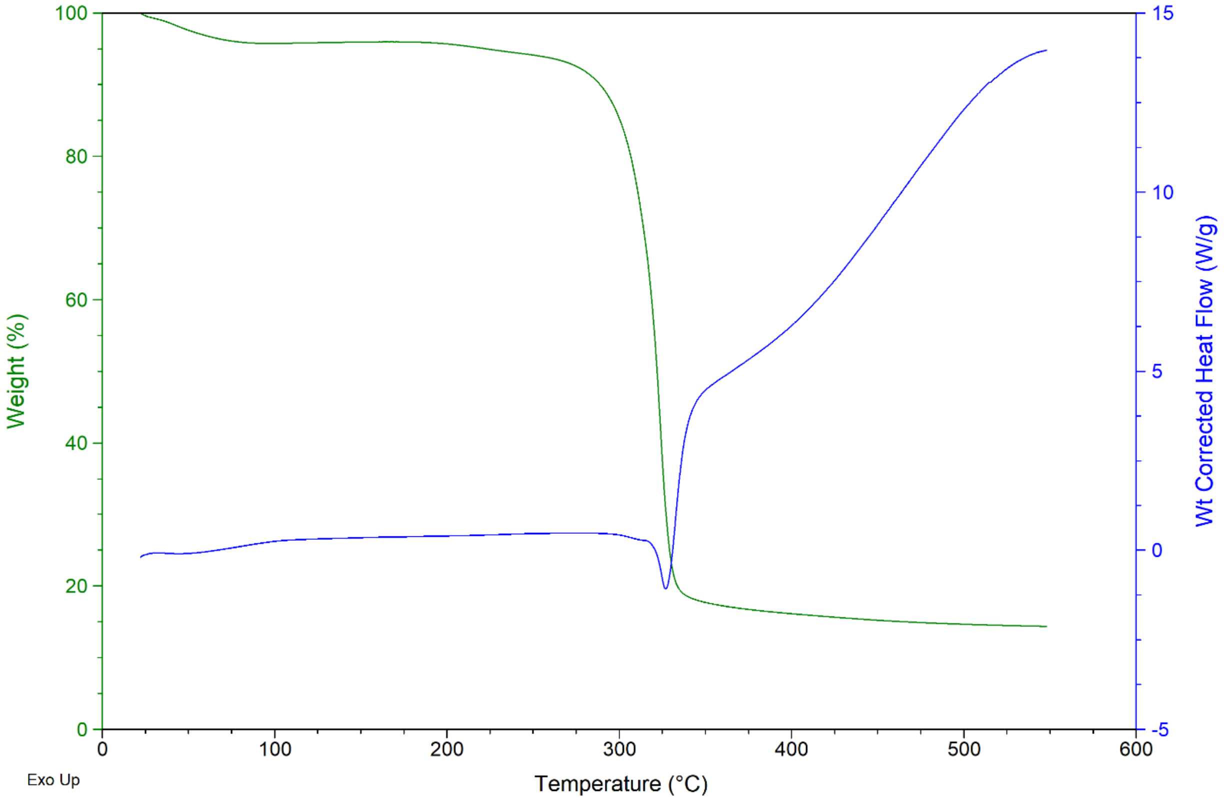

Figure S2: DSC/TGA curve of LYS.

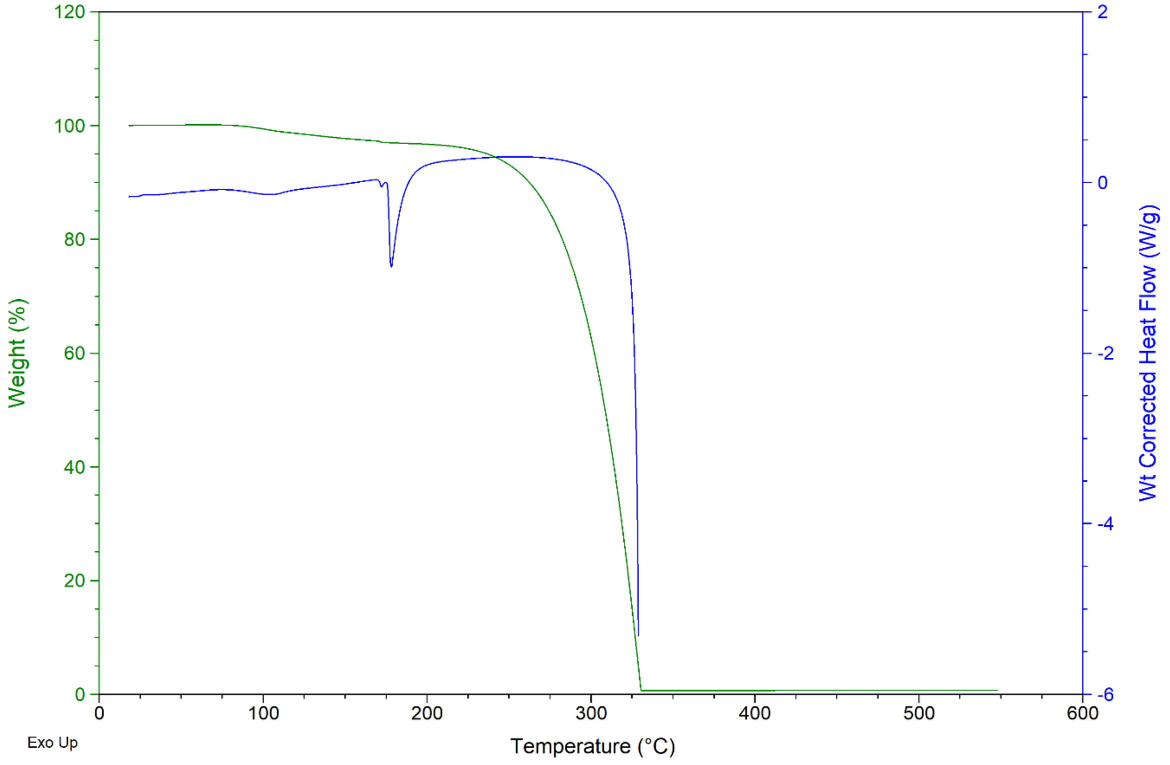

Figure S3: DSC/TGA curve of EST.

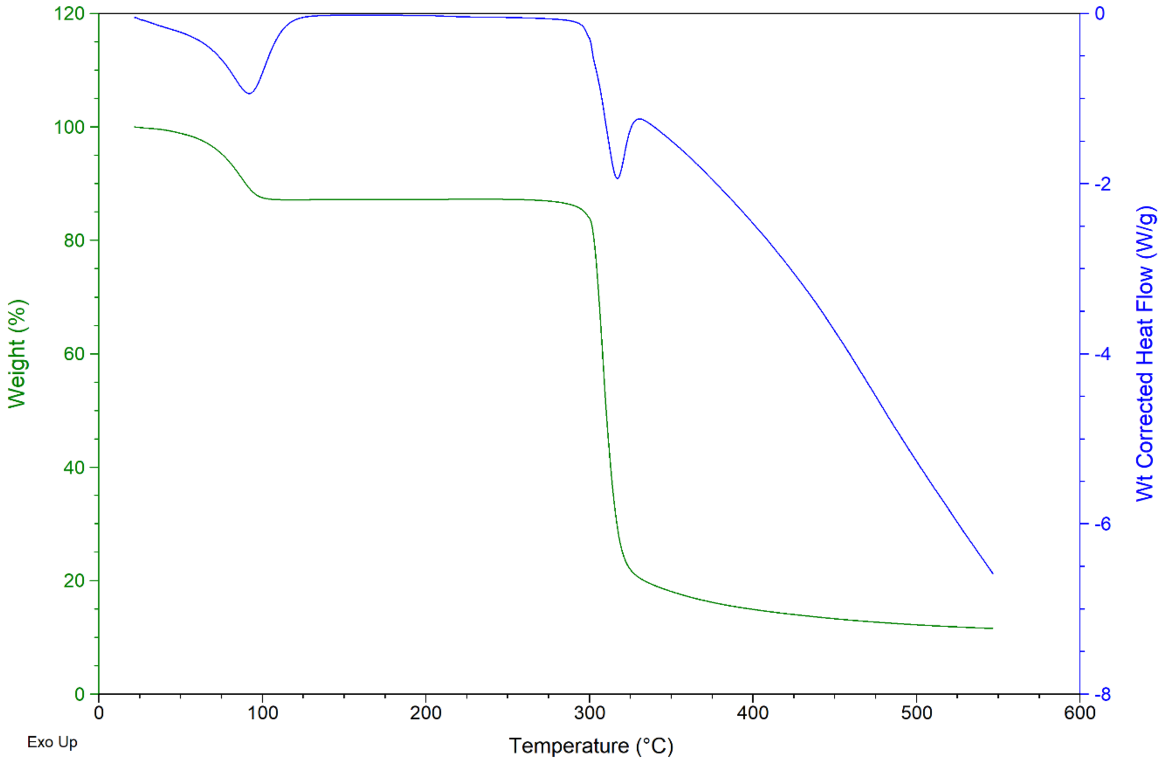

Figure S4: DSC/TGA curve of β-CD.

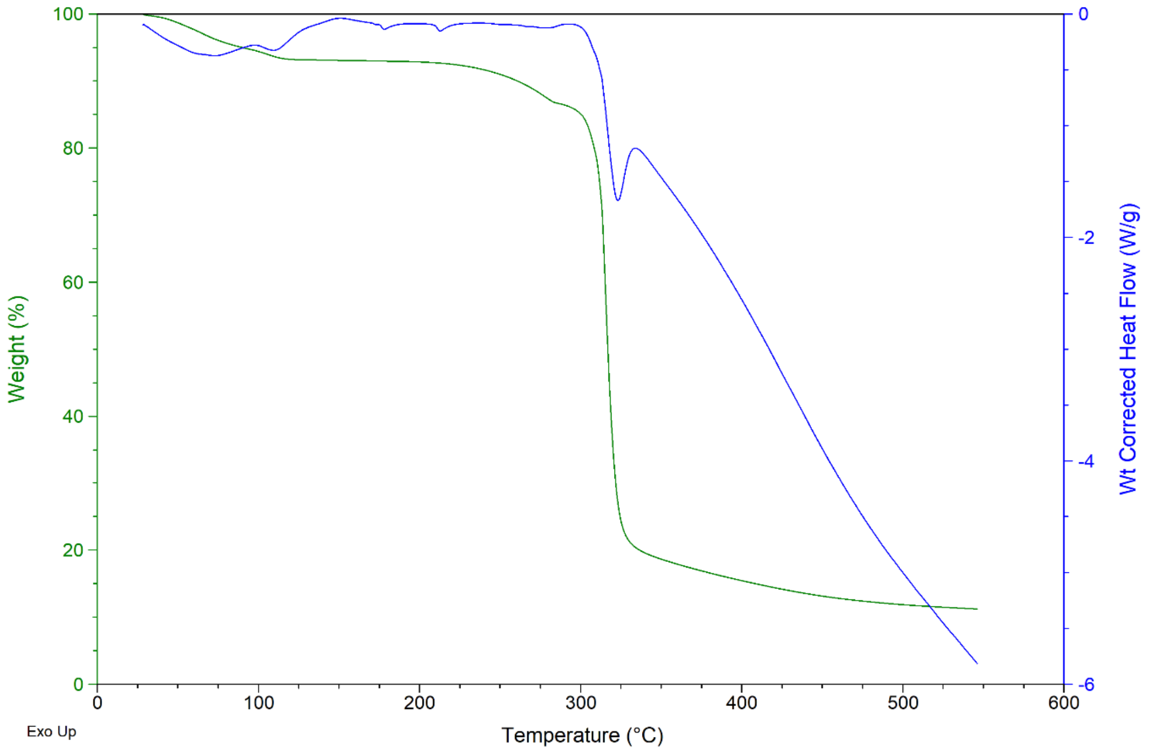

Figure S5: DSC/TGA curve of STAND.

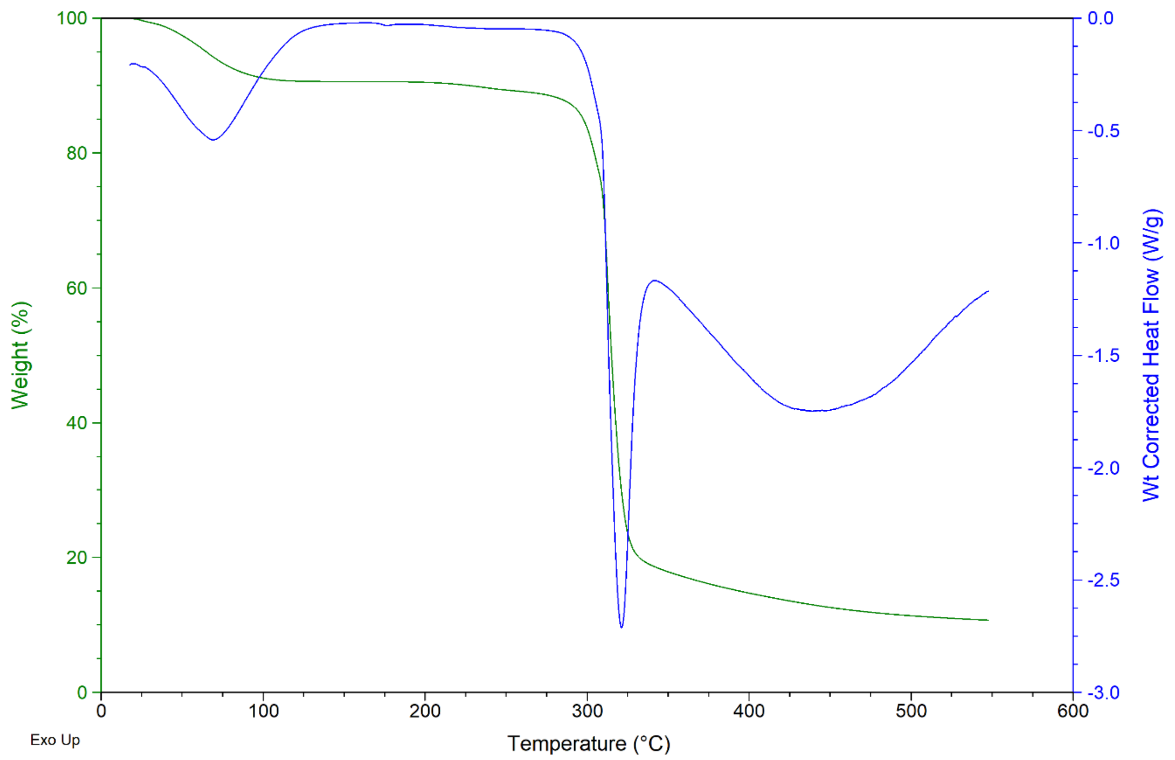

Figure S6: DSC/TGA curve of MECH.

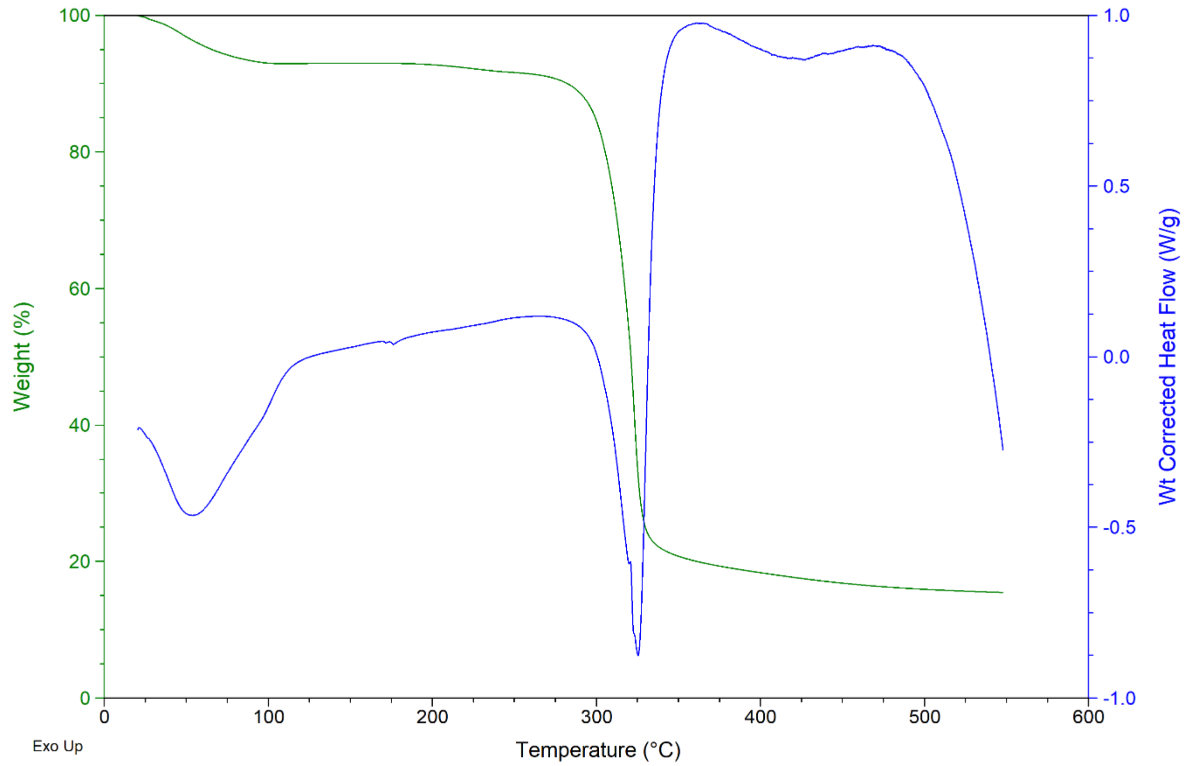

Figure S7: DSC/TGA curve of STANDSHORT.
